# Tract Specific Reproducibility of Tractography Based Morphology and Diffusion Metrics

**DOI:** 10.1371/journal.pone.0034125

**Published:** 2012-04-02

**Authors:** René M. H. Besseling, Jacobus F. A. Jansen, Geke M. Overvliet, Maarten J. Vaessen, Hilde M. H. Braakman, Paul A. M. Hofman, Albert P. Aldenkamp, Walter H. Backes

**Affiliations:** 1 Department of Radiology, Maastricht University Medical Center (MUMC+), Maastricht, The Netherlands; 2 Department of Neurology, Maastricht University Medical Center (MUMC+), Maastricht, The Netherlands; 3 School for Mental Health and Neuroscience, Maastricht University Medical Center (MUMC+), Maastricht, The Netherlands; 4 Epilepsy Center Kempenhaeghe, Heeze, The Netherlands; Beijing Normal University, Beijing, China

## Abstract

**Introduction:**

The reproducibility of tractography is important to determine its sensitivity to pathological abnormalities. The reproducibility of tract morphology has not yet been systematically studied and the recently developed tractography contrast Tract Density Imaging (TDI) has not yet been assessed at the tract specific level.

**Materials and Methods:**

Diffusion tensor imaging (DTI) and probabilistic constrained spherical deconvolution (CSD) tractography are performed twice in 9 healthy subjects. Tractography is based on common space seed and target regions and performed for several major white matter tracts. Tractograms are converted to tract segmentations and inter-session reproducibility of tract morphology is assessed using Dice similarity coefficient (DSC). The coefficient of variation (COV) and intraclass correlation coefficient (ICC) are calculated of the following tract metrics: fractional anisotropy (FA), apparent diffusion coefficient (ADC), volume, and TDI. Analyses are performed both for proximal (deep white matter) and extended (including subcortical white matter) tract segmentations.

**Results:**

Proximal DSC values were 0.70–0.92. DSC values were 5–10% lower in extended compared to proximal segmentations. COV/ICC values of FA, ADC, volume and TDI were 1–4%/0.65–0.94, 2–4%/0.62–0.94, 3–22%/0.53–0.96 and 8–31%/0.48–0.70, respectively, with the lower COV and higher ICC values found in the proximal segmentations.

**Conclusion:**

For all investigated metrics, reproducibility depended on the segmented tract. FA and ADC had relatively low COV and relatively high ICC, indicating clinical potential. Volume had higher COV but its moderate to high ICC values in most tracts still suggest subject-differentiating power. Tract TDI had high COV and relatively low ICC, which reflects unfavorable reproducibility.

## Introduction

Diffusion weighted MRI (DWI) can be used to probe tissue water diffusion in vivo and thus can provide unique information on tissue microstructure. It is routinely used in the clinic to assess the extent of lesions in cerebral infarction [1]. However, its full potential is in the unveiling of the directional dependence of diffusion in white matter, which is strongest in the direction of the axonal fibers. This anisotropic diffusion process is often modeled using the diffusion tensor (DT, [2]) and several clinically relevant metrics can be calculated from it, such as fractional anisotropy (FA), which is a measure for the directional coherence of the tracts, and apparent diffusion coefficient (ADC), which is a measure of water motility.

For a variety of neurological diseases, distributed white matter FA decreases and ADC increases have been reported, both indicative of loss of microstructural integrity. Examples include stroke and multiple sclerosis [3] and epilepsy [4].

Since diffusion tensor imaging (DTI) provides voxelwise estimates of fiber orientation, a natural extension is to extrapolate the local orientations to continuous streamlines, representing fiber tracts. This technique is called tractography and opens opportunities for interrogating specific white matter tracts. For example, tractography has been used to segment the pyramidal tract in early-stage multiple sclerosis. It was found that only in case of clinical motor symptoms, the fraction of sclerotic lesions was significantly increased in the pyramidal tract compared to the rest of the brain [5]. Another application is epilepsy, in which tractography of the optic radiation may aid the prediction and prevention of post-operative visual impairment in temporal lobe epilepsy [6].

**Figure 1:**
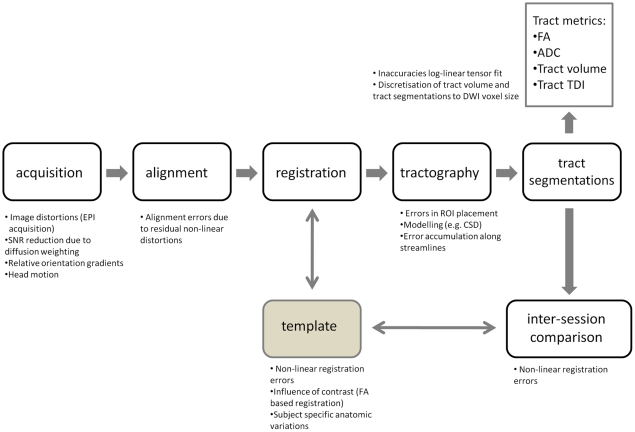
Tractography pipeline. The tractography pipeline employed in this paper, from data acquisition to tract segmentations and tract metrics. At each step, several potential error sources are given.

The tractography pipeline involves many steps, among others data alignment, registration and modeling (fiber orientation and propagation), see Figure 1. These steps all have characteristic sources of error, the combination of which leads to a certain amount of variability in the quantitative end results. In line with this, scan-rescan stability and inter-subject variability of tractography are important measures to determine its potential in revealing pathological abnormalities and changes over time. Few studies have investigated the reproducibility of tractography. These studies mainly focus on tract volume and tensor derived metrics [7], [8], [9], but investigate morphological reproducibility only to a limited extent [9]. Tract morphology involves both tract shape and its embedding within the brain and as such is more descriptive than tract volume. Tract morphology is important in surgical planning, but also in longitudinal studies of cortical remodeling after stroke or injury, which presently are restricted to dye tracer studies in monkeys [10] or gray matter morphological analyses in structural scans [11].

It is known that compared to deep white matter, directly subcortical white matter shows increased bending, fanning and crossing of tracts, which gives rise to partial volume effects. Partial volume effects are inherent to DWI, which assesses microstructure at the meso scale of the imaging voxel, and may compromise tractography accuracy. To address this problem, a multitude of models have been developed which, unlike the diffusion tensor, can represent multiple orientations. These models include q-space imaging (QSI) [12], Q-ball [13], and constrained spherical deconvolution (CSD) [14]. However, to our knowledge, the difference in tractography performance for proximal (i.e. deep white matter) and distal (i.e. directly subcortical) parts of tracts has not been investigated yet.

FA and ADC are voxel metrics and as such are restricted to the DWI imaging resolution. Tract density imaging (TDI) has recently been introduced and provides outstanding anatomical contrast in the white matter [15]. In TDI, a whole brain tractogram is calculated. This tractogram is converted to a tract density map (by counting the number of streamlines per volume element) on a grid which can be much finer than the acquisition resolution of DWI (super resolution), a concept which previously was not considered [16]. The rationale is that in tractography, directional information is integrated over multiple voxels and the resulting increase in consistency allows for super resolution. Whole brain reproducibility of TDI has recently been addressed [17], but not on a tract specific basis.

The purpose of this study is to investigate for several selected well-known tracts both the reproducibility of tract morphology and quantitative metrics. The difference in tractography performance for proximal and distal white matter will also be studied. Tractography will be performed using probabilistic CSD. In addition to conventional tensor metrics (FA and ADC) and tract volume, tract TDI will be investigated as well. The use of non-linear registrations to a common space will be demonstrated for both defining tractography seed and target regions of interest (ROI) and for performing inter-session morphological comparison.

## Materials and Methods

### Subjects

Nine healthy volunteers (6 male, 3 female, age (mean±SD): 28±6 year) were recruited and scanned at Epilepsy Center Kempenhaeghe. None of the subjects had (a history of) neurological or psychiatric disorders or anatomical abnormalities on structural MRI.

### Ethics Statement

This study was approved by the local medical ethical committee of Epilepsy Center Kempenhaeghe. All participants gave written informed consent prior to study participation.

### Data Acquisition

Diffusion weighted MRI (DWI) was performed on a 3 Tesla MRI system (Philips Achieva, maximum gradient strength 40 mT/m, maximum slew rate 200 mT/m/msec) using an 8-element SENSE head coils for parallel imaging (SENSE-factor 2). The imaging resolution was 2×2×2 mm^3^ and a b-value of 1200 s/mm^2^ was used. An echo planar imaging (EPI) sequence was used with echo time (TE) 72 ms and repetition time (TR) 6965 ms. A set of 128 gradient directions was used, optimized via electrostatic repulsion to ensure homogenous distribution over the sphere [18]. A single non-diffusion weighted scan (b0-scan) was obtained. The DWI acquisition time was 15 minutes.

For anatomical reference, a 1×1×1 mm^3^ T1-weighted scan was acquired with TR/TE = 8.1/3.7 ms, inversion time (TI) 1022 ms (3D TFE acquisition, SENSE-factor 1.5), and an acquisition time of 8.5 minutes.

A complete rescan was performed within a couple of weeks (19±18 days) for inter-session comparison.

### Data Preprocessing

Each DWI dataset was aligned to its b0-scan (SNR 20.8±2.6) using affine registrations to correct for patient motion and EPI distortions. This alignment was performed in CATNAP (Coregistration, Adjustment, and Tensor-solving, a Nicely Automated Program, version 3.21) and included correction of the gradient table for the rotations [19], [20]. CATNAP makes use of software routines from FSL (FMRIB software library, Oxford).

### Tractography

All DT and CSD analyses as well as the tractography and the tract segmentations were performed using the MRtrix software package (Brain Research Institute, Melbourne, Australia, http://www.brain.org.au/software/). Registrations to common space were performed in FSL. Additional analyses were performed in Matlab (The MathWorks, Natick, USA).

DT fits were performed to calculate the FA and ADC maps. In addition, fiber orientation distributions (FODs), representing local fiber orientation, were estimated using CSD. In CSD, the diffusion profile is deconvolved with a so called response function, which is the typical diffusion profile of a voxel containing fibers in a single coherent direction. The resulting initial FOD estimate is constrained to suppress noise-induced negative fiber orientations, which leads to the final FOD [14].

For each DWI dataset the CSD response function was estimated from the data. This was done by taking the signal from high FA voxels (FA>0.7) and aligning them based on their first DT eigenvector. This allows subsequent averaging (noise reduction) so a more robust and representative response function can be estimated than would be possible from a single (arbitrary) high FA voxel.

In CSD, FODs are represented by spherical harmonics, which form a basis for functions over the sphere, much like the Fourier series forms a basis for functions over Cartesian space [21]. In agreement with [22], the spherical harmonics order was taken to be no higher than l_max_ = 8 to limit overfitting of noise. This corresponds to 45 spherical harmonics.

Probabilistic tractography was performed using FOD sampling [23]. In this method, the tract propagation direction is selected from the FOD using a sample rejection scheme adhering to both a curvature constraint and an amplitude threshold. MRtrix default settings were used, which include stepsize 0.2 mm, maximum curvature radius 1 mm and FOD amplitude threshold 0.1. The tractogram of each selected major white matter tract (see below) consisted of 10,000 streamlines.

### Selected Tracts

**Figure 2:**
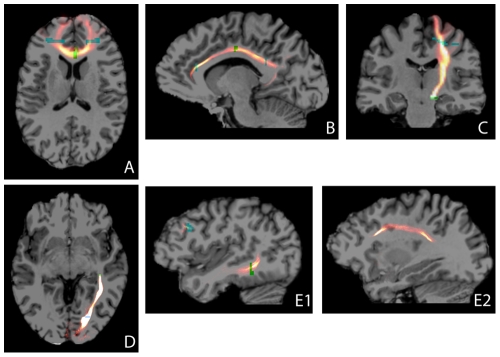
Tractography examples. Tract density maps of a representative subject. Seed ROIs are given in green, target ROIs in blue. For each subfigure, the tract density windowing (yellow-red) is chosen such that subcortical projections can also be appreciated. The skull-stripped T1 underlay was registered to the diffusion space (b0-scan) using an affine transformation. A: genu of corpus callosum; B: cingulum; C: pyramidal tract; D: optic radiation; E1, E2: arcuate fasciculus (different sagittal slices).

Tractography was performed for a number of well known major white matter tracts of various orientations, locations, and size, see Figure 2. These tracts provide different tractographical challenges.

#### Genu of the corpus callosum

The corpus callosum (CC, Figure 2A) is the largest bundle of the brain and forms the major commissure connecting both hemispheres. Because of its commissural nature and the high directional coherence of its medial part, tractography seed regions are easily defined, although it is difficult and somewhat arbitrary to define distal target regions that limit the extent of the tractogram. The genu of the CC connects the prefrontal and orbitofrontal regions [24].

#### Cingulum

The cingulum (Ci, Figure 2B) is a major associative bundle that is located within the cingulated gyrus. It runs over and around the corpus callosum and below the corona radiata, both of which are likely locations for unwanted spurious streamlines. The Ci contains fibers of different length, the longest of which runs from the anterior temporal gyrus to the orbitofrontal cortex [24].

#### Pyramidal tract

The pyramidal tract (PT, Figure 2C) is a descending projection bundle of fibers from the motor cortex to the brainstem. The PT crosses the CC and the Ci, among others.

#### Optic radiation

The optic radiations (OR, Figure 2D) are large, heavily myelinated projection bundles that consist of fibers primarily between the lateral geniculate nuclei of the thalamus and the primary visual cortices at the bases of the calcarine sulci in the occipital lobe [6]. Proper tractographical reconstruction of the OR is complicated by the sharp turn it makes in Meyer’s loop [6].

#### Arcuate fasciculus

The arcuate fasciculus (AF, Figures 2E) is a lateral associative bundle composed of long and short fibers connecting the perisylvian cortex of the frontal, parietal, and temporal lobes. The AF of the left hemisphere is involved in language [24]. It is one of the four constituents of the superior longitudinal fasciculus (SLF) [25] and tractographical delineation is especially challenging.

### Tractography ROI Placement

Binary tractography ROIs were defined in FSLs 1×1×1 mm^3^ average FA space. Tract specific ROIs were defined in single axial, sagittal, or coronal slices, depending on the tract orientation. ROIs were subsequently dilated using a 3×3×3 voxel kernel, giving them a final thickness of 3 mm. Two ROI types were used: seed ROIs, from which streamlines were started, and target ROIs, which streamlines must reach to be included in the final tractogram.

For the genu of the CC, the Ci, the PT, and the OR, the ROI placement guidelines of [9] were followed. For the AF, the guidelines of [8] for SLF delineation were adapted to additionally include the AF-specific temporal projections.

ROIs were registered to each dataset using FSLs non-linear registration and the associated parameter set optimized for alignment to FSL’s average FA space (http://www.fmrib.ox.ac.uk/fsl/fnirt/). This scheme ensures objective, operator-independent mapping of the common space ROIs to each dataset. This registration step is given by the vertical double headed arrow in Figure 1. After mapping to native space, ROIs were re-binarized using a threshold of 0.01 to correct for interpolation effects.

### Proximal and Extended Tractograms

**Figure 3:**
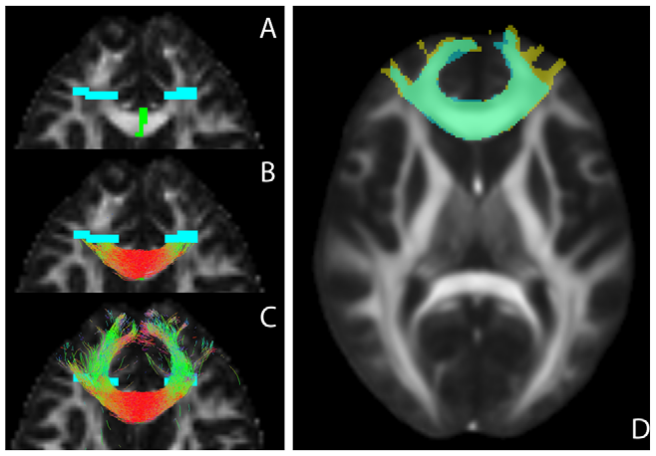
Proximal and extended tract segmentations and inter-session comparison. A: ROIs for the genu of the corpus callosum overlaid on the FA map, seed ROI in green, target ROIs in blue; B: proximal tractogram; C: extended tractogram. D: tract segmentations of both scan sessions of the same subject, registered to the common FA space (underlay). Segmentation 1 in blue, 2 in yellow and segmentation overlap in green.

Tractography was performed both excluding and including cortical projections. In the first case (proximal), streamlines were propagated until they reached the target ROIs (Figure 3B). In the second case (extended), streamlines were allowed to propagate beyond the target ROIs until they reach the edge of the brain mask (Figure 3C).

### Registration of Tract Segmentations

Tractograms were converted to tract density maps at the DWI acquisition resolution by counting the number of streamlines per voxel. These tract density maps were thresholded at 5% of their maximum value to construct binary tract segmentations.

Inter-session morphological comparison of tract segmentations was performed in common space, see the horizontal double headed arrow in Figure 1. Also for within-subject comparison, mapping of tract segmentations to a common space is desirable because of scan-specific image distortions inherent to the DWI sequence. FSLs average FA space was used for both subject-independent ROI definition (see above) and as session-independent common space for all diffusion based maps (FA, ADC, TDI), see the vertical and horizontal arrow, respectively, in Figure 1. Native space tract segmentations were mapped to the common space by reversing the non-linear deformation field for native space ROI construction, see Figure 3D.

After mapping to common space, tract segmentations were re-binarized using a threshold of 0.01.

### Tract Metrics

The tract segmentations at the DWI resolution were also used to select the relevant voxels for calculating mean tract FA and ADC in the native space. Furthermore, they were used to calculate native space tract volume.

### Tract Density Imaging

Whole brain tractography was performed using 5,000,000 tracts seeded from random locations within the white matter. For each subject, the white matter was segmented from the T1 image and mapped to the diffusion spaces of both sessions via affine registrations to the b0-scans.

Whole brain tractograms were converted to whole brain tract density images (TDIs) by counting the number of streamlines per element on a 1 mm grid. It is emphasized that this is not merely an interpolation step. In TDI, voxel-wise (local) information on fiber orientation is extrapolated to continuous (global) streamlines using tractography. Streamline continuity and smoothness (ensured by tractography constraints such as maximum curvature) are assumed to yield spatial consistency at a higher level of detail. This allows performing TDI at a grid size small than the acquisition resolution. Since the DWI acquisition resolution was 2 mm, performing TDI at 1 mm provides limited super resolution at the benefit of an acceptable number of streamlines (data storage).

Tract TDI was assessed by constructing tract segmentations at the TDI grid size. For each tract, the TDI elements within this segmentation were used to calculate mean tract TDI. Similar to the calculation of tract segmentations at the DWI resolution, a threshold of 5% of the maximum value was used to convert tract probability maps to binary tract segmentations.

In addition to assessing TDI at the tract level, a whole brain TDI atlas was constructed. For this, each whole brain TDI needed to be mapped to the common space, i.e. FSLs average FA space. For each dataset, the coordinates of all the streamlines were mapped to the common space using the nonlinear deformation field obtained in FA normalization [17]. Subsequently subject specific normalized TDIs were calculated and combined to construct a whole brain TDI atlas.

### Reproducibility Measures

#### Dice similarity coefficient

Inter-session morphological agreement of common space tract segmentations was quantified using the Dice similarity coefficient [26]:
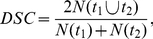
(1)in which t_1_ and t_2_ stand for the tract segmentations of session 1 and 2, respectively, and N(t_i_) gives the number of voxels. DSC varies between 0 and 1 for no and complete spatial overlap, respectively.

#### Coefficient of variation

The reproducibility of tract metrics was examined using the coefficient of variation (COV), which is defined as
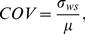
(2)with σ_ws_ the within-subject standard deviation of the measure of interest and µ its population mean [27]. The COV is a measure for the precision of a measure and gives an indication of the minimum detectable relative deviation from the mean.

#### Intraclass correlation coefficient

To disentangle the sources of variation, the intraclass correlation coefficient (ICC) was calculated:
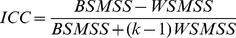
(3)with BSMSS the between-subject mean sum of squares, WSMSS the within-subject mean sum of squares and k the number of scans per subject [28]. The ICC ranges from -1 (no reliability, that is, BSMSS = 0) to 1 (maximum reliability, achieved in the case of identity between test and retest, that is, WSMSS = 0) [29]. The higher a metric’s ICC, the better it reflects between-subject differences and the higher its clinical potential.

## Results

### Tract Segmentation Overlap

**Figure 4:**
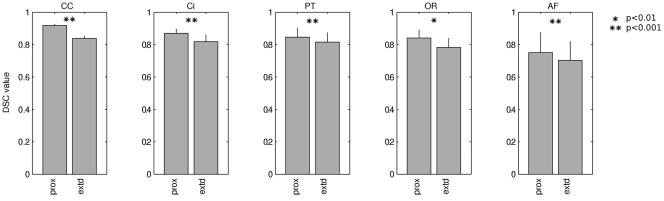
Morphological reproducibility. Inter-session spatial overlap of tract segmentations, quantified by Dice similarity coefficient (DSC). Results are shown for both proximal (prox) and extended (extd) segmentation. Error bars represent 1 standard deviation. CC: genu of the corpus callosum; Ci: cingulum; PT: pyramidal tract; OR: optic radiation; AF: arcuate fasciculus.

In Figure 4, mean inter-session overlap as quantified by DSC (Equation 1) is shown for both proximal and extended tract segmentations. Compared to proximal overlap, extended overlap was 5%–10% lower in all cases. Inter-tract differences are clearly visible, with the genu of the CC showing most inter-session overlap and the AF showing least. DSC ICC values were 0.75–0.92 for the proximal segmentations and 0.70–0.84 for the extended segmentations (σ_bs_ = 0.007–0.123 and 0.013–0.115, respectively). Complete results are provided in Table 1.

**Table 1:** Morphological reproducibility.

DSC: between session	proximal		extended		p-value
segmentation overlap	mean	σbs	mean	σbs	
corpus callosum	0.92	0.007	0.84	0.013	<0.0005
Cingulum	0.87	0.026	0.82	0.041	<0.0005
pyramidal tract	0.85	0.055	0.82	0.058	<0.0005
optic radiation	0.84	0.048	0.78	0.054	0.002
arcuate fasciculus	0.75	0.123	0.70	0.115	0.001

Summary statistics for the inter-session overlap of tract segmentations, quantified by DSC. The p-values are of a paired t-test for difference between proximal and extended values. σ_bs_: between-subject standard deviation.

### Tract Metrics

**Figure 5:**
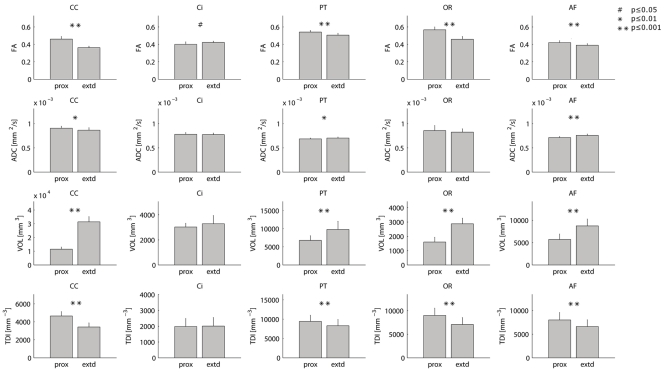
Reproducibility of tract metrics. Tract metrics for both proximal (prox) and extended (extd) tract segmentations. The p-values are of paired t-tests for difference between proximal and extended values. FA: fractional anisotropy; ADC: apparent diffusion coefficient; VOL: tract volume; TDI: tract density. CC: genu of the corpus callosum; Ci: cingulum; PT: pyramidal tract; OR: optic radiation; AF: arcuate fasciculus.

The tract metrics are visualized in Figure 5. Quantitative results are given in Table 2. Bland-Altman plots are provided in Figures S1 and S2 and show that the precision of each metric is independent of the metric value. For the OR, the statistics were based on the data from 8 subjects as for one subject the first session DWI brain coverage was incomplete.

**Table 2:** Reproducibility of tract metrics.

A										
extended	FA					ADC [×10–3 mm2/s]				
	mean	σws	σbs	COV	ICC	mean	σws	σbs	COV	ICC
CC	0.362	0.010	0.015	0.028	0.635	0.864	0.014	0.052	0.016	0.932
cing	0.421	0.015	0.011	0.035	0.109	0.773	0.016	0.031	0.021	0.754
PT	0.506	0.015	0.021	0.029	0.600	0.703	0.013	0.019	0.018	0.617
OR	0.458	0.009	0.032	0.019	0.926	0.824	0.017	0.068	0.021	0.938
AF	0.389	0.005	0.019	0.014	0.921	0.757	0.015	0.028	0.020	0.754

	Vol [ml]					TDI [×103]				
	mean	σws	σbs	COV	ICC	mean	σws	σbs	COV	ICC
	31.364	1.557	3.629	0.050	0.831	3.424	0.296	0.412	0.087	0.589
	3.284	0.391	0.590	0.119	0.641	2.007	0.620	0.333	0.309	-0.268
	9.799	1.573	2.019	0.161	0.534	8.304	1.216	1.447	0.146	0.478
	2.879	0.190	0.379	0.066	0.776	7.070	1.551	1.002	0.219	-0.090
	8.732	1.682	1.127	0.193	-0.054	6.610	0.834	1.286	0.126	0.653

B										
proximal	FA					ADC [×10–3 mm2/s]				
	mean	σws	σbs	COV	ICC	mean	σws	σbs	COV	ICC
CC	0.460	0.008	0.031	0.016	0.942	0.906	0.017	0.041	0.018	0.845
cing	0.400	0.011	0.028	0.027	0.866	0.780	0.019	0.037	0.024	0.770
PT	0.541	0.011	0.017	0.021	0.653	0.685	0.010	0.016	0.015	0.659
OR	0.567	0.010	0.032	0.018	0.910	0.855	0.030	0.105	0.035	0.920
AF	0.420	0.010	0.021	0.024	0.786	0.712	0.011	0.022	0.015	0.790

	Vol [ml]					TDI [×103]				
	mean	σws	σbs	COV	ICC	mean	σws	σbs	COV	ICC
	11.468	0.313	1.574	0.027	0.961	4.643	0.357	0.449	0.077	0.521
	3.026	0.173	0.276	0.057	0.669	1.982	0.539	0.344	0.272	-0.103
	6.775	0.794	1.192	0.117	0.637	9.421	1.045	1.428	0.111	0.578
	1.603	0.098	0.334	0.061	0.917	9.007	1.705	1.081	0.189	-0.109
	5.704	1.224	0.891	0.215	0.029	7.999	0.873	1.467	0.109	0.699

C										
p-value	FA	ADC	Vol	TDI						
CC	<0.0005	0.005	<0.0005	<0.0005						
cing	0.028	0.113	0.218	0.734						
PT	<0.0005	0.006	<0.0005	0.001						
OR	<0.0005	0.067	<0.0005	<0.0005						
AF	<0.0005	<0.0005	<0.0005	<0.0005						

Reproducibility of tract metrics for both proximal (A) and extended (B) tract segmentations and paired t-test p-values for comparison of proximal and extended metrics (C). σ_bs_: between-subject standard deviation; σ_ws_: within-subject standard deviation; COV: coefficient of variation; ICC: intraclass correlation coefficient.

FA: fractional anisotropy; ADC: apparent diffusion coefficient; VOL: tract volume; TDI: tract density.

CC: genu of the corpus callosum; Ci: cingulum; PT: pyramidal tract; OR: optic radiation; AF: arcuate fasciculus.

#### Fractional anisotropy

In all tracts except the Ci, the FA values were significantly higher for the proximal tract segmentations. For the Ci, no difference was found. Note that the Ci is the only tract studied that has no extensive cortical projections. Furthermore, for the Ci the volumes of the extended and the proximal segmentations were not significantly different. In the proximal case the FA COV was 2–3%, in the extended case this was 1–4%. The proximal FA ICC value range was 0.65–0.94 and about the same range was found in the extended case, except for a low value in the Ci (0.11).

#### Apparent diffusion coefficient

For the ADC, slightly but significantly higher values were found in the proximal segmentations for the genu of the CC. On the other hand, in the PT and the AF, the proximal values were slightly but significantly lower. In the OR and the Ci, no significant differences were found. The following values were found: COV 2–4% (proximal) and 2% (extended), ICC 0.66–0.92 (proximal) and 0.62–0.94 (extended). These values were in the same range as those for FA.

#### Tract volume

In all tracts except the Ci, the volumes of the extended segmentations were significantly larger than those of the proximal segmentations. For the Ci, no difference was found. COVs were 3–22% (proximal) and 5–19% (extended). Highest values were found in the AF, for which also very low ICC values were found, 0.029 (proximal), and −0.054 (extended). For the other tracts, the ICC values ranges were 0.64–0.96 (proximal) and 0.53–0.83 (extended).

#### Tract TDI

The TDI values were significantly higher for the proximal segmentations in all tracts except for the Ci, in which no significant difference was found. COV values were in the range 8–28% (proximal) and 9–31% (extended). No reliable ICC estimates could be found for the Ci and the OR (−0.268 and −0.090, respectively). The ICC values for the other tracts were 0.52–0.70 (proximal) and 0.48–0.65 (extended).

### TDI Atlas

**Figure 6:**
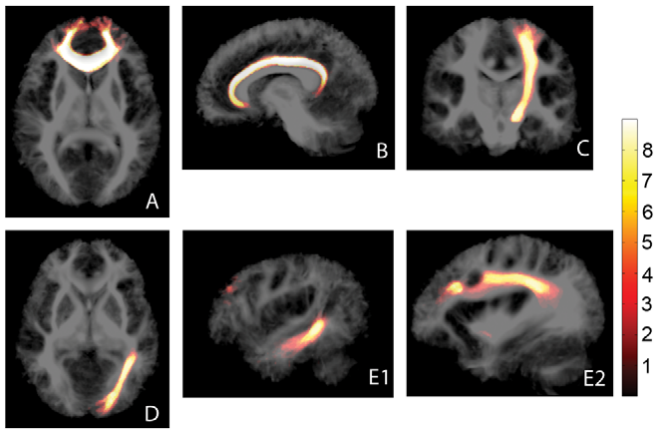
TDI atlas. Average TDI map, depicted in logarithmic scale to enhance subcortical contrast. Overlaid are the summed common space tract segmentations. Colorbar: number of subjects (1–9). Note that reduced tract density at the subject level (Figure 2) coincides with low between-subject segmentation overlap at the group level (directly subcortical regions). A: genu of corpus callosum; B: cingulum; C: pyramidal tract; D: optic radiation; E1, E2: arcuate fasciculus (different sagittal slices).

The TDI atlas is shown in Figure 6. For reference, the common space tract segmentations of all subjects were added and overlaid, providing group segmentation probability maps. Figure 6 shows that TDI provides clear white matter contrast and is high in large, well-known bundles and low in subcortical regions. These subcortical regions do not necessarily have lower fiber densities, but might exhibit larger within-subject variability and/or lower tractography precision.

## Discussion

In this paper, we studied reproducibility of tract morphology and of conventional tract tensor metrics FA and ADC, tract volume, and TDI. We used a common space (FSLs 1mm iso FA space) to both define tractography ROIs and to calculate inter-session tract segmentation overlap. We demonstrated that reproducibility of both segmentation overlap and quantitative measures is strongly tract-dependent. We also found lower reproducibility for subcortically extended compared to deep white matter metrics.

### Tract Segmentation Overlap

We have reported inter-session segmentation overlaps of DSC = 0.67–0.92. In the study of [9], within-subject reproducibility of tract morphology was investigated over 3 sessions by linear registration to the first session. Only the average DSC value over subjects and tracts was reported (0.81), which falls within the range we found. We also reported that segmentation overlap differs between tracts. Especially in the AF, reduced overlap was observed (DSC = 0.67–0.75). We propose that this is related to the complex anatomy of the AF, which is one of the 4 subcomponents of the SLF [25], and for which it difficult to define precluding tractography ROIs. Suboptimal ROI placement in combination with minor registration errors may have led to partial volume effects causing incomplete or non-exclusive segmentation of the AF. Even though guidelines from the literature were followed for common space ROI definition [8], investigation of inter-operator variability should be a subject of future research. Tractograms can also be improved by using a more sophisticated scheme to construct session-specific ROIs [6], but this is beyond the scope of this paper.

We demonstrated that the inclusion of subcortical regions reduces the segmentation overlap (5–10% lower DSC values in extended segmentations compared to proximal segmentations). This is indicative of reduced tractography performance directly below the cortex, which reveals an important limitation of the technique. Potential causes for sub-optimal performance are increased partial volume effects caused by increased fanning, bending and crossing, and error accumulation because of increased distance from the tractography seed. Both effects cannot be disentangled because of the lack of a golden standard (true tract anatomy). In an attempt to reduce the influence of partial volume effects, we used a model that can deal with multiple fiber orientations per voxel (CSD). Error accumulation along the tract, however, is inherent to any streamline tractography algorithm since streamline propagation is an iterative process. This error accumulation can only be avoided by using algorithms that are not streamline based. Examples of such algorithms include fast marching tractography [30] and Bayesian techniques [31]. However, streamline tractography is still the method of choice in the majority of DWI studies.

### Tract Metrics

#### Fractional anisotropy

Significantly decreased FA values were found for the extended segmentations of tracts with cortical projections. A loss of directional coherence (fanning, bending) might explain the trend of decreased FA values in proximity of the cortex. Since tractography was constrained to a brain mask, extended segmentations may contain gray matter, which also decreased mean FA. This effect will be limited because of the FOD amplitude threshold of 0.1, which limits tractography to regions where fiber direction is well-defined (primarily white matter).

The FA COV values found for extended segmentations are comparable to those in [9] and better than those in [7] (6.2%, 18.6% and 7.1% compared to 2.8%, 2.9% and 1.9% in the current study for the CC, the PT and the OR, respectively). Possible explanations for the improvement in performance are the higher field strength of 3T (1.5T in [9] and [7]) and differences in tractography ROI definition. In [7], manually placed single voxel ROIs were used and in [9], linear (affine) registration was used to map common space ROIs to native space, as opposed to non-linear registration in the current study. Another difference is that both other studies used DT based tractography, whereas we used CSD. Except for the extended segmentations of the Ci, the low COVs combined with moderate to high ICC values, 0.65–0.94 (proximal) and 0.60–0.93 (extended), which is indicative of the clinical potential of FA in revealing pathological differences. The low ICC values for the extended segmentations of the Ci are caused by relatively large within-subject variability (compared to between-subject variability) since in some subjects the distal streamlines enter into regions of low FA for one session only. Careful tuning of the tractography parameters might circumvent this problem, however, this might lead to suboptimal performance in other tracts. For the sake of consistency we chose to keep tractography algorithm and parameter settings constant. However, we stress that tractography performance can be improved by optimizing tractography parameters to the tract under investigation. For example, the curvature threshold we used yielded good results but was too conservative to reconstruct Myers’ loop in the OR, see Figure 2 D. It is known from the literature that segmentation of Myers’ loop is very challenging and requires a dedicated approach [6].

#### Apparent diffusion coefficient

For ADC, the COV and ICC values were in the same range as those for FA. The proximal COV values are 1.5–3.5% and the extended COV values are 1.6–2.1%, which is comparable to 1.49–2.14% as found in [9]. The ICC range is 0.66–0.92 (proximal) and 0.62–0.94 (extended). Unlike for FA, for ADC no deviant Ci ICC values were found, probably because ADC displays less contrast than FA in many brain regions. It is known that at the relatively low clinical b-value of b = 1200 s/mm^2^ we employed, even ADC differences between gray matter and white matter are relatively small [32]. This reduces the influence of inter-session differences in the extent of distal streamlines.

#### Tract volume

Extended tract segmentations trivially have a larger volume than proximal tract segmentations because of the additional inclusion of cortical projections. Compared to FA and ADC, the COV value of tract volume is relatively high. Proximal values are 3–20% and extended values are 5–19%, which is comparable to literature values of 8.15–13.03% [9]. Except for the AF, the ICC values, on the other hand, indicate moderate to good subject-differentiating power (0.64–0.96 (proximal) and 0.53–0.83 (extended)). This underlines that inter-session reproducibility should be seen relative to between-subject variability. The poor performance in the AF is probably caused by minor scan-specific registration errors combined with suboptimal ROI placement, which leads to relatively large inter-session differences as discussed previously.

#### Tract TDI

We observed decreased TDI values for the extended tract segmentations. This is likely caused by a combination of increased fanning and bending in directly subcortical regions and error accumulation along the streamlines which leads to distal streamline divergence.

The TDI COV can be quite high (9–30% for extended) and is comparable to a literature value of 26% (WM average, [17]). In addition, ICC estimates are low (negative in OR and Cing, for both proximal and extended segmentations). This suggests that in the current approach, the between-subject differentiating power of tract TDI is low. It has recently been shown that an extension on TDI, average pathlength mapping (APM), has better COV (WM average of 19%, [17]). APM does not only consider the number of streamlines, but also the streamline length, and might have better clinical potential [17], although the biological interpretation is different.

### Clinical Implications

Reproducibility should be seen in the light of pathological abnormalities to make statements about sensitivity. In brain DWI, the literature on pathological differences in FA and ADC is extensive. For example, in epilepsy with malformations of cortical development, local pathological differences in FA and ADC have been reported, both in gray and in white matter. Compared to the values in healthy controls, FA reductions down to 51% and ADC increases up to 119% have been reported [4]. These differences are large with respect to the COV values we report here, however, this was in patients with active epilepsy and obvious structural lesions. A more subtle example is a study about post-operative seizure-free temporal lobe epilepsy, in which regions of reduced FA were found both within and outside the temporal lobe [33]. This study indicates that FA can be used to assess loss of microstructural integrity after seizure remission and also outside the seizure onset zone (temporal lobe), which might be of relevance for follow-up or the unveiling of diseased networks. The average FA reduction of the affected regions was 0.09 (approximately 15%), which is still relatively large compared to the COV values we report.

Most DWI studies, however, only report the significance of the changes in FA and ADC and do not report the effect size. For this reason, several DWI reproducibility studies reverse the question and provide power analyses that, given a certain effect size, e.g. 10%, provide the number of measurements needed to achieve sufficient statistical power [8], [9]. In agreement with our results, these studies report comparable sensitivities for FA and ADC (on the order of 10 subjects per group) and a much lower sensitivity for volume (100 subjects per group).

It is important to realize that reproducibility and anatomical plausibility of tractography are mere surrogates for the technique’s accuracy since true tract anatomy is unknown at the subject level. The pipeline from data acquisition to tract segmentations is very long in tractography, with many and diverse potential error sources (Figure 1). The high DSC values we report suggest that at least the morphology of the structures we reconstruct is reproducible. Since tract tensor metrics and volume are derived from the tract segmentation itself, high morphological reproducibility is assumed to be a prerequisite for high reproducibility of these derived metrics. However, from the relatively low DSC values for the AF it is expected that if the reconstructed morphology does not fully translate to a single anatomical structure, but instead is incomplete or too extensive, reproducibility is reduced.

Finally, the number of subjects in this study (9) was limited but comparable to that in other reproducibility studies [7], [8], [9]. To investigate the effect of group size on reproducibility, we performed bootstrap analysis, taking subsets from our original 9 subject group. This showed that the relative increase in reproducibility achieved by recruiting an additional subject beyond around 10 is relatively low compared to adding a subject at smaller group sizes (results not shown). However, we expect the main source of uncertainty to be within-subject and since within-subject reproducibility is a key issue in individual diagnosis and longitudinal studies, we suggest increasing the number of scans per subject instead of the group size for future research.

### Conclusion

We demonstrated that tractography based tract segmentations can be used to assess tract morphology in a reproducible manner in addition to their more typical use as ROIs to investigate tract metrics such as tract FA, ADC, and volume. We demonstrated that metric reproducibility is strongly tract dependent and also depends on whether or not directly subcortical projections are included (proximal versus extended tract segmentations). Generally speaking, in our approach FA and ADC both show good COV and good ICC, which indicates good reproducibility and good subject-differentiating power, respectively. For tract volume, higher COV values were found, indicative of lower reproducibility, but since the ICC values were still reasonable, subject differentiation was preserved. For tract TDI, on the other hand, both COV was increased and ICC reduced, indicating both low reproducibility and low subject- differentiating power.

## Supporting Information

Figure S1 **Tract metric Bland-Altman plots for the proximal tract segmentations.** The between-session difference of each metric (y-axis) is independent of the between-session mean of the metric. This indicates that in case of a pathological change of the metric (decrease or increase) within the investigate range, this effect is not obscured by change in precision. However, note that there are differences in precision (y-dispersion) between tracts, indicating reproducibility differences between tracts. FA: fractional anisotropy; ADC: apparent diffusion coefficient; VOL: tract volume; TDI: tract density.CC: genu of the corpus callosum; Ci: cingulum; PT: pyramidal tract; OR: optic radiation; AF: arcuate fasciculus.
(TIF)

Figure S2 **Tract metric Bland-Altman plots for the extended tract segmentations.** The same as Figure S1, but now for the metrics of extended tract segmentations.
(TIF)
