# Supplementary figures and images for: Tract Specific Reproducibility of Tractography Based Morphology and Diffusion Metrics
Source: PLoS One. 2012 Apr 2;7(4):e34125. doi: 10.1371/journal.pone.0034125 (PMC3317780; doi:10.1371/journal.pone.0034125)

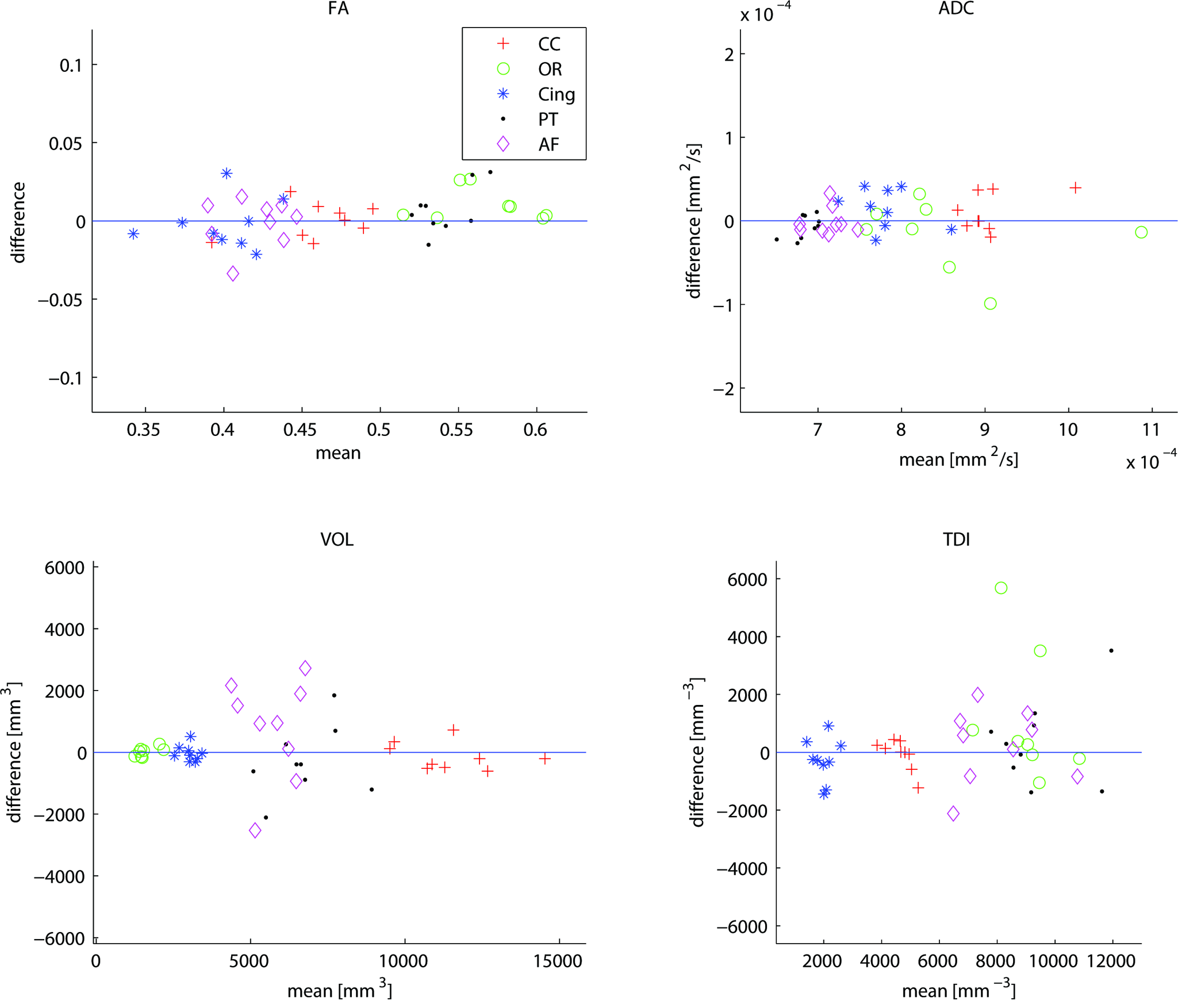

Supplement: Figure S1 — Tract metric Bland-Altman plots for the proximal tract segmentations. The between-session difference of each metric (y-axis) is independent of the between-session mean of the metric. This indicates that in case of a pathological change of the metric (decrease or increase) within the investigate range, this effect is not obscured by change in precision. However, note that there are differences in precision (y-dispersion) between tracts, indicating reproducibility differences between tracts. FA: fractional anisotropy; ADC: apparent diffusion coefficient; VOL: tract volume; TDI: tract density.CC: genu of the corpus callosum; Ci: cingulum; PT: pyramidal tract; OR: optic radiation; AF: arcuate fasciculus. (TIF) [file pone.0034125.s001.tif]

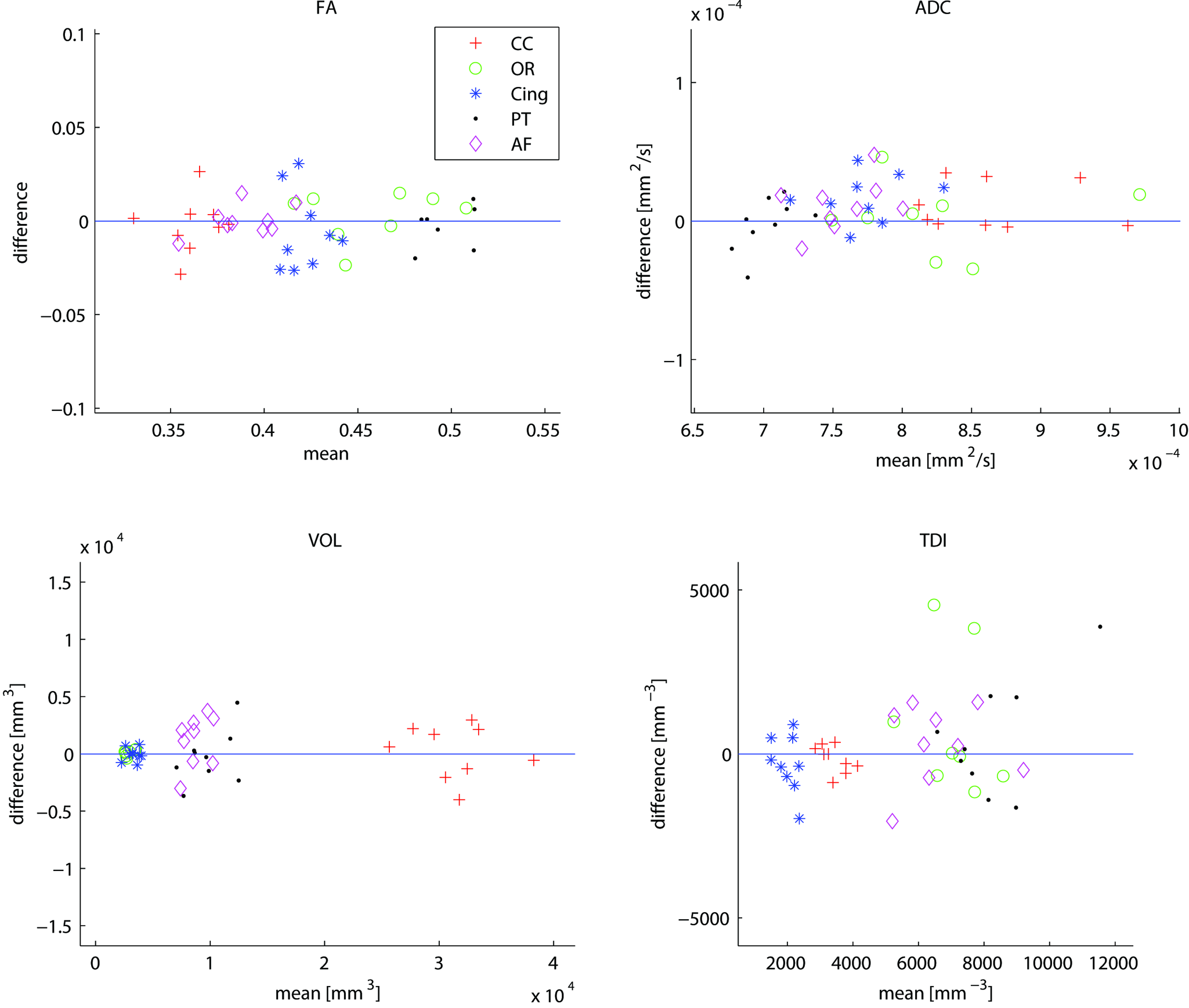

Supplement: Figure S2 — Tract metric Bland-Altman plots for the extended tract segmentations. The same as Figure S1, but now for the metrics of extended tract segmentations. (TIF) [file pone.0034125.s002.tif]
